# The quality and reliability evaluation of Parkinson’s disease-related short videos on social short video platforms: cross-sectional study

**DOI:** 10.1038/s41598-026-50589-w

**Published:** 2026-04-28

**Authors:** You Feng, Lele Liu, Hai Mi, Dan Xu

**Affiliations:** 1 Taicang Hospital of Traditional Chinese Medicine，Taicang TCM Hospital Affiliated to Nanjing University of Chinese Medicine, 140 Renmin South Road, Taicang, 215400 China; 2 Nanjing University of Chinese Medicine, 138 Xianlin Road, Nanjing, 210023 China

**Keywords:** Parkinson’s disease, Short video, TikTok, Bilibili, Information quality, Digital health, Health care, Medical research

## Abstract

Parkinson’s disease represents a major global public health burden, with China projected to have the highest age‑standardized prevalence worldwide. Short video platforms have become important channels for health information dissemination, yet the quality of Parkinson’s disease‑related content on these platforms remains systematically unassessed. This study aimed to evaluate the quality and reliability of Parkinson’s disease‑related short videos on two leading Chinese platforms, TikTok and Bilibili. Using a cross‑sectional design, we collected the top 100 videos from each platform on April 11, 2026. Videos were classified by uploader identity and content theme. Quality was assessed using the JAMA Benchmark Score, a modified DISCERN medical information quality scale (mDISCERN), and the Global Quality Score (GQS). Results showed platform specific differences in uploader composition, content coverage, video length, and engagement. Overall, the videos showed moderate educational quality and reliability, but limited transparency and accountability. TikTok videos scored higher on mDISCERN, whereas JAMA scores did not differ significantly between platforms. Videos uploaded by healthcare professionals generally received higher quality scores. Correlations between engagement and quality were weak and platform dependent. These findings suggest that Parkinson’s disease related short videos vary in quality across platforms, and that popularity is not a reliable indicator of information quality.

**Supplementary Information:**

The online version contains supplementary material available at 10.1038/s41598-026-50589-w.

## Introduction

Parkinson’s disease (PD), as the second most common neurodegenerative disorder worldwide, poses a significant global public health challenge. By 2050, the number of patients is projected to double from 2021 levels to 25.2 million, indicating a substantial increase in disease burden. This burden is unevenly distributed globally, with China projected to bear a particularly heavy share. The country will anticipate approximately 10.5 million cases and possess the world’s highest age-standardized prevalence rate^1^. The disease not only causes progressive motor dysfunction but is also accompanied by multiple non-motor symptoms such as cognitive decline and psychiatric disorders^2–4^. Therefore, these symptoms significantly diminish patients’ quality of life, exacerbate the disease burden, and pose a severe challenge to global public health systems^1,5^.

In this situation, patients, caregivers, and the public increasingly seek reliable disease information. Video platforms, with their visual appeal, high interactivity, and rapid dissemination, have become vital channels for sharing health knowledge^6,7^. Therefore, by translating complex medical concepts into accessible formats, they greatly enhance public access to health information^8^. In China, TikTok and Bilibili have become the two most prominent short video platforms for health communication. TikTok’s powerful recommendation algorithms provide users with highly tailored content, making it a major source of health information for the public. Bilibili offers extensive educational content, including health and medical topics, typically in longer-duration short videos^9^. Collectively, both platforms host a large amount of disease-related content including PD, and serve as crucial health information sources for patients and caregivers in China. Based on the aforementioned characteristics of the two platforms, it is reasonable to infer that in the content ecosystem surrounding PD related short videos, TikTok tends to produce concise, accessible, and algorithmically recommended fragmented short videos, whereas Bilibili is more inclined to publish in depth, systematic, and longer form medical science content. The specific differences between the two platforms, however, remain to be further verified.

However, due to relatively lax content moderation and creators from diverse professional backgrounds, these platforms also host a significant volume of information that lacks scientific verification or is even misleading. Current research on other diseases has similarly revealed that the overall quality of video content on such platforms tends to be suboptimal^10,11^. Based on current assessments of the quality of short video content on neurological disorders, including migraine^12^, depression^13^, and epilepsy^14^, on popular short video platforms, although no direct study has yet focused on PD, existing evidence indicates that the overall quality of condition related short videos fails to meet the “excellent” threshold of the Global Quality Scale (GQS), with most videos ranging from “poor” to “moderate” quality. Among these, videos produced by neurology specialists demonstrate superior reliability and accuracy. Extending the analysis to common diseases such as diabetes^15^, cardiovascular diseases^16,17^, and cancer^18–20^, a dual platform evaluation based on TikTok and Bilibili reveals that video durations on Bilibili are generally two to four times longer than those on TikTok, whereas TikTok videos achieve higher GQS scores. This discrepancy is closely associated with the higher level of professional physician involvement observed on TikTok. Both platforms share three common characteristics regarding video content quality. First, the overall completeness and credibility of the videos remain generally low. Second, the correlation between video quality and user engagement is inconsistent; most studies report a positive correlation, although some show no significant correlation or even a negative one. Third, videos produced by professional physicians are of significantly higher quality, yet remain relatively few in number.

The issue of video quality on Chinese platforms such as TikTok and Bilibili is not an isolated case. Relevant studies have revealed that medical content on international video platforms such as YouTube and Vimeo also suffers from widespread deficiencies in quality. For instance, assessments of videos related to permanent tooth fracture management and piriformis muscle injections on YouTube have shown that in both cases, over 50% of the videos were rated as low quality on the GQS^21,22^. This indicates that the deficiency in the quality of medical video content represents a widespread issue across various mainstream video platforms, with limited association to the geographical location or national affiliation of the platforms. In summary, despite preliminary progress in quality assessments of short video content for various diseases on these platforms, systematic research on PD, a condition with high prevalence and substantial disease burden in China, remains notably absent. Therefore, there is an urgent need to explore the differences in PD related short video content between TikTok and Bilibili, in order to help users better identify and select high quality health information.

Based on the aforementioned research background, this study aims to systematically evaluate the content quality, information accuracy, and scientific reliability of PD-related videos on China’s two major short video platforms, TikTok and Bilibili. Through this research, we hope to provide the audience with a reference for selecting more suitable health-related content, further optimize health communication regarding PD, and offer a basis for developing targeted public health information strategies.

## Methods

### Search strategy and data collection

**Fig. 1:**
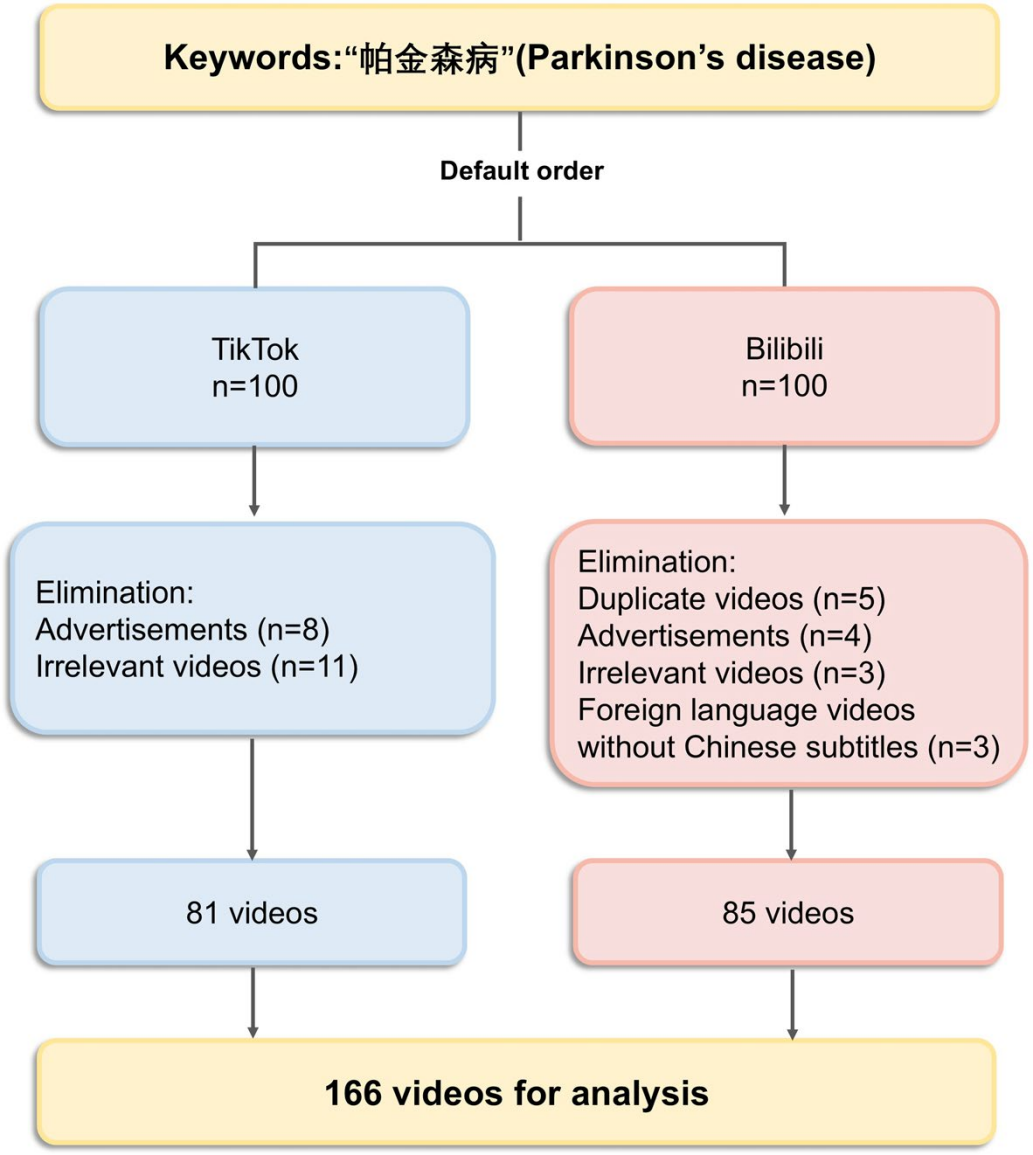
The detailed data acquisition workflow for videos of PD.

To obtain the most recent content, we collected short video data from two major Chinese social media platforms, TikTok and Bilibili within a cross-sectional study framework on April 11, 2026 in Nanjing, Jiangsu Province, China. New accounts were registered on each platform for the aim of minimizing algorithmic bias. Using the Chinese keyword “帕金森病” (PD), we retrieved the top 100 videos under the default order from each platform. Existing evidence demonstrates that videos ranked beyond position 100 exert negligible analytical influence^23^. The retrieved videos were independently screened by 2 reviewers with basic neurological knowledge. Disagreements regarding video inclusion were resolved through discussion, with a third senior researcher consulted when necessary. The initial results were filtered to remove (1) duplicate videos (within each platform); (2) purely commercial advertisement; (3) irrelevant videos; (4) foreign language videos without Chinese subtitles. All remaining eligible videos were included in the final analysis. The detailed data acquisition workflow is summarized in Fig. 1.

### Classification of videos

Following discussion, the videos were categorized from two primary dimensions. First, by author identity. Publishers were classified into four main categories: (1) healthcare professional individuals; (2) healthcare institutions; (3) non-professional individuals; and (4) non-professional organizations. Healthcare professional individuals were subdivided into (1) neurologists; (2) other clinical specialists; (3) allied healthcare professionals; (4) practitioners of Traditional Chinese Medicine (TCM); and (5) medical students. Healthcare institutions were further divided into (1) official healthcare organizations and (2) other medical entities. Non-professional individuals included (1) science communicators and (2) patients and their family members. Non-professional organizations comprised (1) news media and (2) other public service organizations. Second, by core content, videos were thematically categorized into six domains: (1) epidemiology; (2) etiology; (3) diagnosis; (4) treatment; (5) prevention; and (6) prognosis. If a video covers multiple topics, each topic will be documented. The same 2 reviewers independently classified the included videos by author identity and content category. Any disagreements were resolved through discussion or consultation with a third senior researcher. Inter-rater reliability for video selection, author identity classification, and content classification was assessed using Cohen’s kappa, and all kappa values were no lower than 0.750.

### Video quality and reliability assessments

The quality and reliability of the videos were assessed using three validated instruments. Video information quality was evaluated with the Journal of the American Medical Association Benchmark Score (JAMA)^24^, which assesses four dimensions: authority, disclosure, currency, and attribution and yields a total score ranging from 0 to 4 points. Content reliability was measured using a modified DISCERN medical information quality scale (mDISCERN) instrument adapted for short videos which comprises 5 items: aims and achievement, source reliability, balance and unbiasedness, additional references, uncertainty disclosure, resulting in a total score between 0 and 5 points^25,26^.Overall presentation and usefulness were rated with the Global Quality Score (GQS), a 5-point scale where 1 indicates very poor quality and 5 indicates excellent quality^27^. The detailed scoring criteria for JAMA, mDISCERN, and GQS are presented in Supplementary Tables 1–3. Two independent raters, both possessing basic medical knowledge of neurology and having completed standardized training on all scoring criteria, performed the assessments. The sequence in which videos were reviewed was randomized to prevent order bias. Differences in scoring were discussed and resolved jointly, with input from a third senior researcher when required. Inter-rater reliability for the rating results was assessed using the intraclass correlation coefficient (ICC) based on a two-way mixed-effects model with absolute agreement. All ICC values were > 0.810. Detailed Cohen’s kappa and ICC results are provided in Supplementary Table 4.

### Statistical analyses

All statistical analyses were performed using GraphPad Prism software (version 10.1.2) and the software of R (version 4.1.3). These outcome measures are ordinal in nature. The Shapiro-Wilk test confirmed that the distributions of all score sets significantly deviated from normality. Consequently, descriptive statistics are presented as median and interquartile range. For group comparisons, the Mann-Whitney U test was used for comparisons between 2 independent groups, and the Kruskal-Wallis test was employed for comparisons involving more than 2 groups. When the Kruskal-Wallis test indicated a statistically significant difference, Dunn’s post-hoc test was applied for pairwise comparisons. Furthermore, Spearman’s rank correlation analysis was used to examine the associations between objective video characteristics and each of the quality scores. To account for multiple testing, *p* values from the correlation analyses were adjusted using the false discovery rate (FDR) method with the Benjamini-Hochberg procedure (BH). The *p* value of less than 0.05 was considered statistically significant for all tests.

## Results

### Video characteristics

**Fig. 2:**
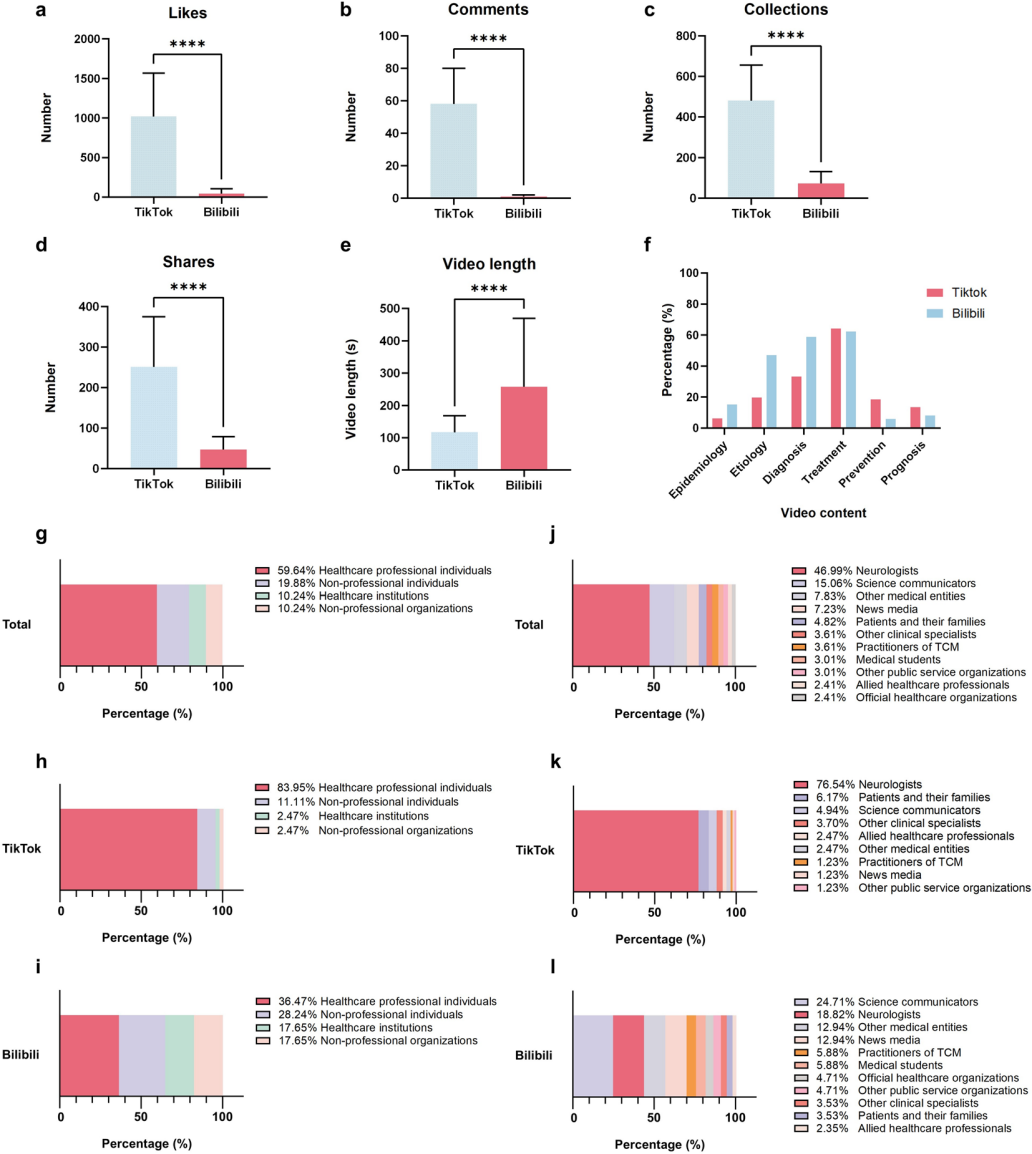
Comparative analysis of TikTok and Bilibili regarding engagement metrics, content types and creator identities. (**a**–**e**) A median-based comparison of likes, comments, collections, shares, and video length between TikTok and Bilibili. (**f**) The quantity of videos with different content on TikTok and Bilibili. (**g**–**i**) The distribution of creator identity categories for total dataset, TikTok, and Bilibili. (**j**–**l**) The distribution of detailed creator identity categories for total dataset, TikTok, and Bilibili. ****p < 0.0001.

A total of 166 videos (81 from TikTok and 85 from Bilibili) were included in the final analysis. The general information of short videos from different platforms are listed in Table 1. Distinct platform-specific patterns were observed in terms of user engagement and video duration. Videos on TikTok showed higher user engagement, including the number of likes, comments, and collections, compared to those on Bilibili (Fig. 2a–d) (all *p <* 0.0001). This indicates that PD-related videos on TikTok generated broader audience engagement and stronger dissemination potential. However, videos on Bilibili were significantly longer in length, suggesting that Bilibili may be more likely to host content with more extended and in-depth explanations (Fig. 2e) (*p* < 0.0001).

**Table 1:** The general information of videos from different platforms.

Variable	Total	TikTok	Bilibili
Basic information			
Likes, M (IQR)	305 (1388.75)	1019 (2351)	46 (157)
Comments, M (IQR)	10.5 (65.5)	58 (169)	1 (3)
Collections, M (IQR)	190.5 (628)	480 (1014)	73 (192)
Shares, M (IQR)	113.5 (351.5)	251 (739)	47 (174)
Video length, M (IQR)	171.5 (285)	117 (140)	258 (984)
Quality			
JAMA score, M (IQR)	2 (0)	2 (0)	2 (1)
GQS score, M (IQR)	3 (1)	3 (0)	3 (1)
mDISCERN score, M (IQR)	3 (2)	3 (1)	2 (1)
Video content			
Epidemiology, N (%)	18 (10.8)	5 (6.2)	13 (15.3)
Etiology, N (%)	56 (33.7)	16 (19.8)	40 (47.1)
Diagnosis, N (%)	77 (46.4)	27 (33.3)	50 (58.8)
Treatment, N (%)	105 (63.3)	52 (64.2)	53 (62.4)
Prevention, N (%)	20 (12.0)	15 (18.5)	5 (5.9)
Prognosis, N (%)	18 (10.8)	11 (13.6)	7 (8.2)
Video source			
Healthcare professional individuals, N (%)	99 (59.6)	62 (84.0)	31 (36.5)
Neurologists, N (%)	78 (47.0)	62 (76.5)	16 (18.8)
Other clinical specialists, N (%)	6 (3.6)	3 (3.7)	3 (3.5)
Allied healthcare professionals, N (%)	4 (2.4)	2 (2.5)	2 (2.4)
Practitioners of TCM, N (%)	6 (3.6)	1 (1.2)	5 (5.9)
Medical students, N (%)	5 (3.0)	0 (0.0)	5 (5.9)
Healthcare institutions, N (%)	17 (10.2)	2 (2.5)	15 (17.6)
Official healthcare organizations, N (%)	4 (2.4)	0 (0.0)	4 (4.7)
Other medical entities, N (%)	13 (7.8)	2 (2.5)	11 (12.9)
Non-professsional individuals, N (%)	33 (19.9)	9 (11.1)	24 (28.2)
Science communicators, N (%)	25 (15.1)	4 (4.9)	21 (24.7)
Patients and their families, N (%)	8 (4.8)	5 (6.2)	3 (3.5)
Non-professional organizations, N (%)	17(10.2)	2 (2.5)	15 (17.6)
News media, N (%)	12 (7.2)	1 (1.2)	11 (12.9)
Other public service organizations, N (%)	5 (3.0)	1 (1.2)	4 (4.7)

The themes covered in videos across both platforms were similar, encompassing all core domains. But Bilibili included a greater proportion of videos addressing specialized topics such as epidemiology, etiology, diagnosis, and treatment compared with TikTok (Fig. 2f). This suggests that Bilibili tends to provide more detailed and knowledge-oriented medical explanations, whereas TikTok may place greater emphasis on concise and highly engaging content delivery.

Analysis of uploader identity by broad category showed marked differences between the two platforms. On TikTok, videos were predominantly uploaded by healthcare professional individuals (*N* = 62, 84.0%), indicating a relatively concentrated source of medical information (Fig. 2h). By contrast, Bilibili demonstrated a more diverse uploader composition, with contributions distributed across healthcare professional individuals (*N* = 31, 36.5%), non-professional individuals (*N* = 24, 28.2%), healthcare institutions (*N* = 15, 17.6%), and non-professional organizations (*N* = 15, 17.6%) (Fig. 2i). Overall, healthcare professional individuals (*N* = 99, 59.6%) and non-professional individuals (*N* = 33, 19.9%) represented the two main uploader groups, while healthcare institutions (*N* = 17, 10.2%) and non-professional organizations (*N* = 17, 10.2%) accounted for comparatively smaller proportions (Fig. 2g). These findings suggest that TikTok content in this field is more strongly driven by individual medical professionals, whereas Bilibili reflects a more diverse source composition.

Regarding detailed uploader identity (Fig. 2j), neurologists (*N* = 78, 47.0%) constituted the largest proportion of content creators overall, underscoring their central role in the dissemination of information related to PD on short video platforms. Other uploader types, including science communicators (*N* = 25, 15.1%), other medical entities (*N* = 13, 7.8%), news media (*N* = 12, 7.2%), patients and their families (*N* = 8, 4.8%), practitioners of TCM (*N* = 6, 3.6%), and medical students (*N* = 5, 3.0%), contributed smaller proportions. Official healthcare organizations (*N* = 4, 2.4%) and allied healthcare professionals (*N* = 4, 2.4%) were among the least represented categories overall.

A platform-specific breakdown revealed further distinctions (Fig. 2k, l). On TikTok, neurologists remained the predominant uploader group, with all other uploader categories accounting for only a small proportion. In contrast, Bilibili showed a more diverse pattern, with science communicators representing the largest uploader type, followed by neurologists and other medical-related contributors. Compared with TikTok, Bilibili also included a broader range of uploader identities, including the medical students and the official healthcare organizations.

Taken together, these findings indicate that TikTok videos were associated with higher levels of user engagement and shorter duration, with uploader identity concentrated predominantly among neurologists. In contrast, Bilibili videos were longer and were uploaded by a more diverse range of uploader types while covering a wider range of specialized topics.

### Video quality and reliability assessments

**Fig. 3:**
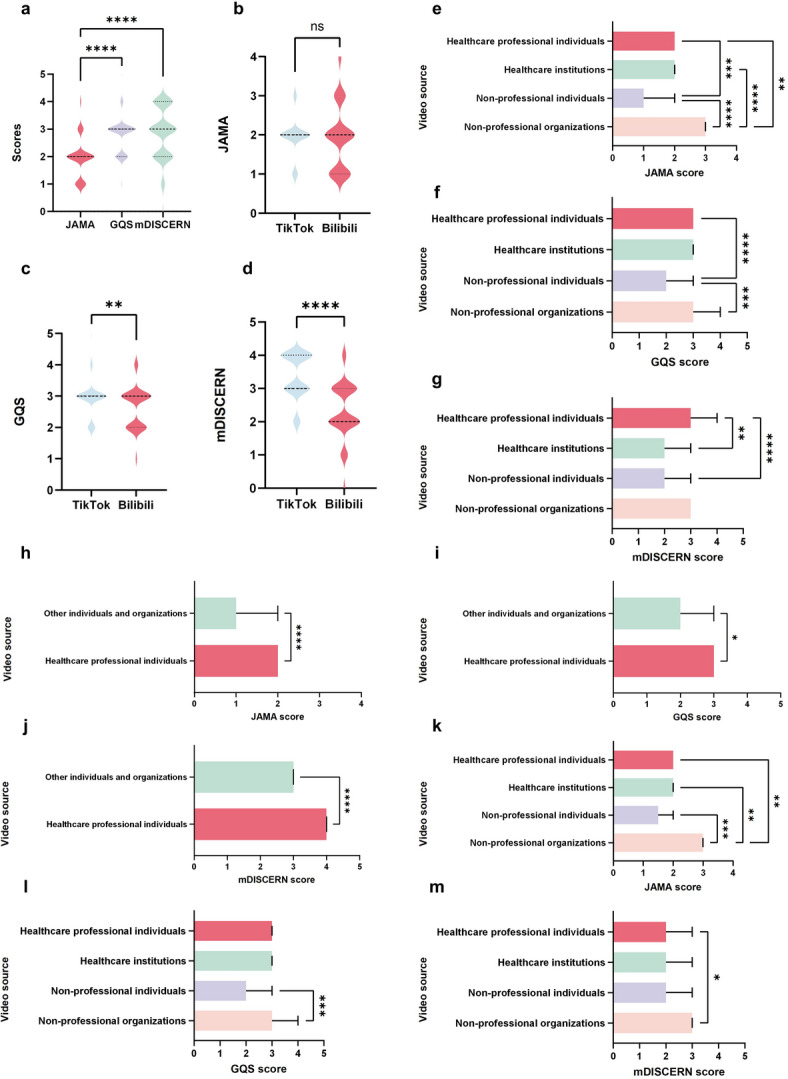
A comparative analysis of video quality between platforms and among video sources. (**a**) The overall status of JAMA, GQS, mDISCERN scores. (**b**–**d**) Comparison of JAMA, GQS, mDISCERN scores between TikTok and Bilibili. (**e**–**g**) Bar charts of the scores for JAMA, GQS, and mDISCERN across different identity categories. (**h**-**j**) Bar charts of the scores for JAMA, GQS, and mDISCERN across different identity categories on TikTok. (**k**-**m**) Bar charts of the scores for JAMA, GQS, and mDISCERN across different identity categories on Bilibili. ns: not significant,  *p* > 0.05; *p < 0.05; **p < 0.01; ***p < 0.001; ****p < 0.0001.

The three evaluation instruments yielded distinct scoring distributions (Fig. 3a). The median GQS (IQR 2–3) and mDISCERN (IQR 2–4) scores were both 3, whereas the median JAMA score (IQR 2–2) was lower at 2. Overall, these findings indicate that the included videos generally reached a moderate level of overall quality and information reliability, but performed less well on source transparency and accountability related criteria assessed by JAMA (*p* < 0.0001). Notably, the absence of dispersion in JAMA scores suggests that such limitations were consistently observed across the sampled videos, whereas the broader distribution of mDISCERN scores indicates greater heterogeneity in the reliability of the medical information presented.

At the platform level, no significant difference was observed between TikTok and Bilibili in JAMA scores (Fig. 3b). For GQS, although both platforms had a median score of 3, the distributions differed significantly, with TikTok (IQR 3–3) scores being more tightly clustered and Bilibili (IQR 2–3) scores showing greater dispersion (Fig. 3c) (*p* < 0.01). For mDISCERN, TikTok had a higher median score than Bilibili, at 3 (IQR 3–4) and 2 (IQR 2–3), respectively, indicating a higher overall level of information reliability in TikTok videos (Fig. 3d) (*p* < 0.0001).

Overall, uploader category was associated with differences in JAMA, GQS, and mDISCERN scores. For JAMA, healthcare professional individuals scored higher than non-professional individuals (*p* < 0.001), while non-professional organizations scored higher than healthcare professional individuals (*p* < 0.01), healthcare institutions (*p* < 0.0001), and non-professional individuals (*p* < 0.0001) (Fig. 3e). For GQS, non-professional individuals scored lower than both healthcare professional individuals (*p* < 0.0001) and non-professional organizations (*p* < 0.001) (Fig. 3f). For mDISCERN, healthcare professional individuals scored higher than healthcare institutions (*p* < 0.01) and non-professional individuals (*p* < 0.0001) (Fig. 3g).

Taken together, Fig. 3 shows that evaluation outcomes varied not only between platforms but also across uploader categories, highlighting the influence of both platform characteristics and source identity on video quality and information reliability.

### Correlation analysis

**Fig. 4:**
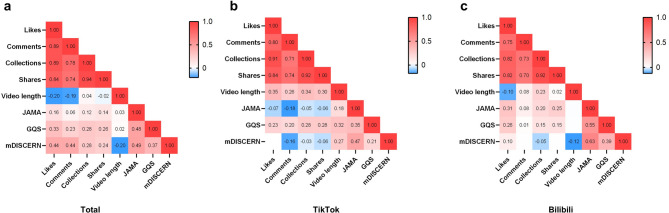
Heatmaps of correlations among video general information for the total dataset, TikTok, and Bilibili.

To examine the relationship between user engagement and video information quality, Spearman correlation analyses were conducted for the overall sample and separately for the TikTok and Bilibili datasets, with *p* values adjusted using the BH. The adjusted *p* values are presented in Tables 2, 3 and 4. Engagement metrics were positively correlated with one another across all datasets, as expected. More importantly, in the overall sample (Fig. 4a), several engagement indicators showed weak to moderate positive correlations with video quality related scores. Specifically, mDISCERN scores were positively correlated with likes (*ρ* = 0.44, *p* < 0.0001), comments (*ρ* = 0.44, *p* < 0.0001), collections (*ρ* = 0.28, *p* < 0.001), and shares (*ρ* = 0.24, *p* < 0.01). GQS scores were also positively correlated with likes (*ρ* = 0.33, *p* < 0.0001), comments (*ρ* = 0.23, *p* < 0.01), collections (*ρ* = 0.28, *p* < 0.001), and shares (*ρ* = 0.26, *p* < 0.01). In addition, JAMA scores were positively correlated with GQS scores (*ρ* = 0.48, *p* < 0.0001) and mDISCERN scores (*ρ* = 0.49, *p* < 0.0001), while GQS scores were positively correlated with mDISCERN scores (*ρ* = 0.37, *p* < 0.0001). These findings suggest that videos with greater user engagement tended to have somewhat better educational quality and reliability, although the strength of these associations was generally modest.

In the TikTok dataset (Fig. 4b), GQS scores were positively correlated with collections (*ρ* = 0.28, *p* < 0.05) and shares (*ρ* = 0.28, *p* < 0.05). Video duration was also positively correlated with GQS scores (*ρ* = 0.32, *p* < 0.01) and mDISCERN scores (*ρ* = 0.27, *p* < 0.05). In addition, significant positive correlations were observed among the quality assessment tools, including JAMA scores with mDISCERN scores (*ρ* = 0.47, *p* < 0.0001) and JAMA scores with GQS scores (*ρ* = 0.35, *p* < 0.01).

In the Bilibili dataset (Fig. 4c), few robust associations remained between engagement metrics and video quality related scores after correction. Only likes showed a weak positive correlation with JAMA scores (*ρ* = 0.31, *p* < 0.05). By contrast, significant positive correlations were consistently observed among the three quality assessment tools, including JAMA scores with mDISCERN scores (*ρ* = 0.63, *p* < 0.0001), JAMA scores with GQS scores (*ρ* = 0.55, *p* < 0.0001), and GQS scores with mDISCERN scores (*ρ* = 0.39, *p* < 0.001).

Together, these results indicate that the relationship between user engagement and information quality was limited and platform dependent, whereas the three quality scoring tools showed relatively consistent trends in assessing video quality related attributes.

**Table 2:** BH FDR-adjusted *p* values for correlation analyses in the total dataset.

Adjusted *p* value	Likes	Comments	Collections	Shares	Video length	JAMA	GQS	mDISCERN
Likes	——^a^	——	——	——	——	——	——	——
Comments	< 0.0001	——	——	——	——	——	——	——
Collections	< 0.0001	< 0.0001	——	——	——	——	——	——
Shares	< 0.0001	< 0.0001	< 0.0001	——	——	——	——	——
Video length	< 0.05	< 0.0001	= 0.671	= 0.822	——	——	——	——
JAMA	< 0.05	= 0.483	= 0.167	= 0.095	= 0.732	——	——	——
GQS	< 0.0001	< 0.01	< 0.001	< 0.01	= 0.822	< 0.0001	——	——
mDISCERN	< 0.0001	< 0.0001	< 0.001	< 0.01	< 0.05	< 0.0001	< 0.0001	——

Significant at *p* < 0.05; ^a^Not applicable.

**Table 3:** BH FDR-adjusted *p* values for correlation analyses in TikTok videos.

Adjusted *p* value	Likes	Comments	Collections	Shares	Video length	JAMA	GQS	mDISCERN
Likes	——^a^	——	——	——	——	——	——	——
Comments	< 0.0001	——	——	——	——	——	——	——
Collections	< 0.0001	< 0.0001	——	——	——	——	——	——
Shares	< 0.0001	< 0.0001	< 0.0001	——	——	——	——	——
Video length	< 0.01	< 0.05	< 0.01	< 0.05	——	——	——	——
JAMA	= 0.623	= 0.147	= 0.737	= 0.669	= 0.147	——	——	——
GQS	= 0.065	= 0.113	< 0.05	< 0.05	< 0.01	< 0.01	——	——
mDISCERN	= 0.994	= 0.199	= 0.799	= 0.669	< 0.05	< 0.0001	= 0.099	——

Significant at *p* < 0.05; ^a^Not applicable.

**Table 4:** BH FDR-adjusted *p* values for correlation analyses in Bilibili videos.

Adjusted *p* value	Likes	Comments	Collections	Shares	Video length	JAMA	GQS	mDISCERN
Likes	——^a^	——	——	——	——	——	——	——
Comments	< 0.0001	——	——	——	——	——	——	——
Collections	< 0.0001	< 0.0001	——	——	——	——	——	——
Shares	< 0.0001	< 0.0001	< 0.0001	——	——	——	——	——
Video length	= 0.544	= 0.636	= 0.081	= 0.991	——	——	——	——
JAMA	< 0.05	= 0.636	= 0.138	= 0.052	= 0.991	——	——	——
GQS	< 0.05	= 0.991	= 0.298	= 0.298	= 0.991	< 0.0001	——	——
mDISCERN	= 0.544	= 0.991	= 0.825	= 0.991	= 0.442	< 0.0001	< 0.001	——

Significant at *p* < 0.05; ^a^Not applicable.

## Discussion

### Principal findings

This cross-sectional study evaluated PD related short videos on TikTok and Bilibili in terms of content characteristics, uploader identity, information quality, and the association between user engagement and video quality. Several main findings emerged. First, clear platform specific differences were observed in engagement, video duration, uploader composition, and topic coverage. Second, the overall educational quality and reliability of the included videos were moderate, whereas transparency and accountability remained limited, consistent with previous research on PD related video-based health information^28^. Third, video quality differed across uploader categories. Finally, the association between user engagement and information quality was weak and platform dependent, suggesting that popularity is not a reliable indicator of video quality^29,30^.

The observed platform differences may not be attributable to platform characteristics alone. They may also partly reflect differences in uploader composition, since TikTok was dominated by healthcare professional individuals, particularly neurologists, whereas Bilibili included a broader mix of uploader types. This distinction may have shaped not only communication style and topic selection but also the degree of source disclosure, evidentiary support, and overall content consistency.

### Quality of the short videos on PD

The quality assessment showed a consistent pattern across the three instruments. GQS and mDISCERN scores suggested moderate educational quality and information reliability, whereas JAMA scores were lower, indicating limited transparency, attribution, and accountability. This is particularly relevant in PD, where patients and caregivers often seek information on diagnosis, treatment, symptom management, and prognosis. In this context, videos may appear useful while still lacking adequate sourcing or evidentiary support^16^.

This issue may be particularly important in PD. Unlike some conditions with more standardized public health messaging, PD is clinically heterogeneous, and symptom profiles, treatment responses, and disease progression vary substantially across patients. As a result, short video content may oversimplify individualized management issues and increase the risk that viewers generalize partial or context dependent information^28^. Misconceptions regarding symptoms, treatment, and disease progression may affect health beliefs and decision making^31,32^. Although TikTok videos showed higher mDISCERN scores and a more concentrated GQS distribution, longer duration on Bilibili did not necessarily correspond to better quality, suggesting that uploader expertise and communication practices may be more important than video length alone.

More broadly, the moderate quality and reliability but limited transparency observed in the present study appear consistent with patterns reported for online health information in other chronic diseases, where practical usefulness may coexist with insufficient sourcing and accountability. However, PD may be especially vulnerable to this problem because of its long disease course, complex symptom burden, and the individualized nature of treatment and self-management. Similar concerns have also been raised in other neurological conditions, such as Alzheimer’s disease^29^, suggesting that misinformation risk may be particularly relevant in neurodegenerative disorders.

### Correlation between video quality and video characteristics

Engagement metrics were positively correlated with one another across datasets, as expected, but these relationships were not the main focus of the study. More importantly, in the overall sample, several engagement indicators showed weak to moderate positive correlations with GQS and mDISCERN scores, suggesting that videos with greater user engagement tended to have somewhat better educational quality and reliability. However, these associations were limited.

Platform specific analyses showed that the relationship between engagement and quality was not stable across platforms. On TikTok, only a few positive correlations remained between engagement indicators and quality related scores after correction. On Bilibili, very few robust associations were retained, except for a weak positive correlation between likes and JAMA scores. In contrast, significant positive correlations were consistently observed among JAMA, GQS, and mDISCERN scores. Together, these findings indicate that user engagement and information quality overlap only partially, and that popularity should not be equated with credibility^33^.

### Practical significance

For viewers and caregivers, high engagement should not be used as an independent marker of trustworthy PD information^34^. For clinicians, the results support the need for more active guidance regarding how patients interpret short video-based disease information^35^, especially in areas vulnerable to misunderstanding, such as diagnosis, treatment, and prognosis. For content creators and platforms, the findings highlight the need to improve source disclosure, evidence presentation, and accountability, while considering both platform format and uploader composition.

### Strengths and limitations

A major strength of this study is that it combined platform comparison, uploader classification, content analysis, quality assessment, and correlation analysis to provide a relatively comprehensive evaluation of PD related short videos. In addition, the use of multiple established instruments allowed different dimensions of video quality to be assessed in a complementary manner.

Several limitations should also be acknowledged. First, even though we used standardized tools and trained raters, the scoring process inherently involves subjective judgment, and discrepancies may persist even when raters achieve substantial agreement^36^. Second, the cross-sectional design reflects video characteristics at the time of sampling and cannot account for later changes in availability, ranking, or user engagement. In addition, no restriction on publication date was applied, and older videos may therefore have had more time to accumulate engagement metrics. Third, although JAMA, GQS, and mDISCERN are widely used instruments, they were not specifically developed for short video content and cannot directly identify false, misleading, or incomplete disease related statements. This remains particularly challenging in PD, where clinical relevance may vary across patients and practice contexts^31^. Fourth, the rating process did not include movement disorders specialists or patients with Parkinson disease. Although standardized instruments and trained raters were used to support methodological consistency, specialists might have provided more nuanced judgment regarding disease specific accuracy and clinical appropriateness, while patients could have offered complementary perspectives on comprehensibility and practical relevance. Future studies should therefore adopt or develop more specific frameworks for the direct assessment of false, misleading, and incomplete disease related information, incorporate both expert and patient perspectives, and further investigate how video quality shapes audience comprehension and behavior through surveys or experimental approaches.

## Conclusion

In summary, PD related short videos on TikTok and Bilibili showed clear differences in engagement, topic coverage, uploader structure, and quality related characteristics. Overall educational quality and reliability were moderate, whereas transparency and accountability remained limited. The observed differences between platforms appear to reflect not only platform characteristics but also differences in uploader composition. Although greater user engagement was associated with somewhat better quality in the overall sample, the relationship between popularity and information quality was weak and platform dependent. These findings highlight the need to improve the credibility, transparency, and disease specific accuracy of PD information on short video platforms.

## Supplementary Information

Below is the link to the electronic supplementary material.

Supplementary Material 1

## Data Availability

The original data for this study were collected as publicly available content from the platforms TikTok (https://www.douyin.com/) and Bilibili (https://www.bilibili.com/). The analyzed datasets generated during this study are available from the corresponding author on reasonable request.

## Abbreviations

BH: Benjamini-Hochberg procedure
FDR: False discovery rate
GQS: Global quality score
IQR: Interquartile range
JAMA: Journal of the American Medical Association Benchmark Score
mDISCERN: modified DISCERN medical information quality scale
PD: Parkinson’s disease
TCM: Traditional Chinese Medicine
