# Supplementary material for: The quality and reliability evaluation of Parkinson’s disease-related short videos on social short video platforms: cross-sectional study
Source: Sci Rep. 2026 Apr 28;16:19687. doi: 10.1038/s41598-026-50589-w (PMC13315597; doi:10.1038/s41598-026-50589-w)
Supplement: Supplementary file 1 — Supplementary Material 1 [file 41598_2026_50589_MOESM1_ESM.docx]

**Supplementary Table 1.** The Journal of the American Medical Association (JAMA) benchmark criteria.

| Score* | Score component | |
| --- | --- | --- |
| 1 score | Authorship | Author and contributor credentials and their affiliations should be provided. |
| 1 score | Attribution | Clearly lists all copyright information and states references and sources for content. |
| 1 score | Currency | Initial date of posted content and subsequent updates to content should be provided. |
| 1 score | Disclosure | Conflicts of interest, funding, sponsorship, advertising, support, and video ownership  should be fully disclosed. |

Note: *The criteria of each aspect were scored separately, and 1 point for each criterion with a total score of 4 points.

**Supplementary Table 2.** The Modified DISCERN (mDISCERN) quality criteria.

| **Reliability Score** |
| --- |
| 1. Is the video clear, concise, and understandable? |
| 2. Are valid sources cited? |
| 3. Is the content presented balanced and unbiased? |
| 4. Are additional sources of content listed for patient reference? |
| 5. Are areas of uncertainty mentioned? |

Note:*(1 point for answer ‘yes’, 0 point for answer ‘no’)

**Supplementary Table 3.** The Global Quality Score (GQS) quality criteria.

| **Item features** | **Points** |
| --- | --- |
| Poor quality; poor flow of the videos; most information missing; not at all useful for patients | 1 |
| Generally poor quality; some information listed, but many important topics missing; of very limited use to patients | 2 |
| Moderate quality; suboptimal flow; some important adequately discussed, but other information poorly discussed; somewhat useful for patients | 3 |
| Good quality and generally good flow; most of the relevant information listed, but some topics not covered; useful for patients | 4 |
| Excellent quality and flow; very useful for patients | 5 |

**Supplementary Table 4.** Inter-rater reliability analysis.

| **Statistic** | **Total** | **TikTok** | **Bilibili** |
| --- | --- | --- | --- |
| Cohen’s kappa  Video selection  Uploader classification subtype | 0.799  0.839 | 0.778  0.907 | 0.824  0.890 |
| Video content classification  Epidemiology | 0.832 | 0.882 | 0.806 |
| Etiology  Diagnosis  Treatment  Prevention  Prognosis | 0.850  0.806  0.815  0.822  0.848 | 0.960  0.795  0.750  0.790  0.791 | 0.764  0.780  0.874  0.883  0.927 |
| ICC  JAMA score  GQS score  mDISCERN score | 0.895  0.838  0.914 | 0.869  0.847  0.811 | 0.901  0.815  0.902 |

Note: Cohen’s kappa was used for categorical variables and interpreted as slight (0.00–0.20), fair (0.21–0.40), moderate (0.41–0.60), substantial (0.61–0.80), and almost perfect (0.81–1.00) agreement. ICC was calculated using a two-way mixed-effects model with absolute agreement for average measures and interpreted as poor (<0.50), moderate (0.50–0.75), good (0.75–0.90), or excellent (>0.90) reliability.
